# Circulating Irisin and Myostatin as Markers of Muscle Strength and Physical Condition in Elderly Subjects

**DOI:** 10.3389/fphys.2019.00871

**Published:** 2019-07-10

**Authors:** Cristina Planella-Farrugia, Ferran Comas, Mònica Sabater-Masdeu, María Moreno, José María Moreno-Navarrete, Oscar Rovira, Wifredo Ricart, José Manuel Fernández-Real

**Affiliations:** ^1^Department of Diabetes, Endocrinology and Nutrition, Girona Biomedical Research Institute (IdIBGi), CIBEROBN (CB06/03/010) and Carlos III Health Institute (ISCIII), Girona, Spain; ^2^Department of Medicine, University of Girona, Girona, Spain

**Keywords:** exercise, elderly, myokines, protein supplementation, irisin

## Abstract

**Background and objective:**

Aging is a physiological process known to produce changes in body composition, affecting the musculature and leading to decreased muscle strength. Muscle in response to exercise acts as an endocrine organ, producing and releasing myokines such as irisin and myostatin that modulate muscular growth. Here, we aimed to evaluate the effects of low intensity resistance exercise, with or without protein supplementation, on body composition, anthropometric parameters and circulating irisin and myostatin in elderly subjects.

**Methods:**

This is a prospective and controlled clinical trial in which subjects were randomized into 3 groups: (1) control group (*n* = 20), (2) low intensity resistance exercise group (RE) (*n* = 14), and (3) low intensity resistance exercise and nutritional support group (RENS) (*n* = 9). Participants, aged 60–75 years, were studied at baseline and 16 weeks thereafter. Body composition was evaluated through bioelectric impedance. Serum irisin and myostatin was measured using ELISA.

**Results:**

At follow-up, RENS resulted in a significant increase in fat free mass (47.4 ± 7.4 vs. 46.5 ± 7.4, *p* = 0.046), the calf muscle circumference (36.4 ± 1.3 vs. 32.3 ± 4.3, *p* = 0.025), and circulating irisin (3 ± 1.1 vs. 2.6 ± 1.3, *p* = 0.030) compared to baseline. RE resulted in a significant increase in grip strength (17.2 ± 4.6 vs. 15.3 ± 4.6, *p* = 0.011) and irisin (3.1 ± 0.8 vs. 2.4 ± 0.3, *p* = 0.011) and decreased walking speed at different distance (*p* < 0.02). Opposite findings in these parameters were observed in control intervention. In line with these findings, the percent change of calf muscle circumference (*p* = 0.003) and fat free mass (*p* < 0.0001) were significantly increased in RENS compared to control, whereas fat mass (*p* = 0.033) was decreased. Interestingly, in this group, strength was positively correlated with fat free mass (*r* = 0.782, *p* = 0.008), and circulating irisin was significantly decreased in those participants with strength loss at the end of the study (*p* = 0.002). No significant correlation between circulating irisin and myostatin in any group was observed.

**Conclusion:**

Circulating irisin, but not myostatin, constitutes a marker for improved muscular performance in elderly subjects.

## Introduction

Aging of population is rapidly accelerating, with dramatic increase of people older than 65 years and a change in the age structure of worldwide population (Christensen et al., 2009). Aging is a physiologic process characterized by a gradual impairment in many body functions and growing risk of disease (Holloszy, 2000), with a decrease in strength, flexibility, aerobic capacity, and force output due to progressive loss of muscle tissue and free fat mass whereas fat mass tend to increase (Vinciguerra et al., 2010).

Evidence from human studies supports the notion that regular, vigorous aerobic exercise is a useful tool, with a dose-response effect, to improve the overall health status and longevity (Teramoto and Bungum, 2010; Ruiz et al., 2011). Exercise stimulates the release of cytokines with autocrine, paracrine and endocrine functions produced in skeletal muscle, termed myokines. Thus, skeletal muscle can be classified as endocrine organ. Irisin and myostatin are myokines involved in adaptations to regular training such as increased muscle mass or muscle hypertrophy. Irisin seems a positive regulator whereas myostatin inhibits muscle growth (Kalinkovichs and Livshi, 2015).

Irisin is the result of proteolytic cleavage product of the fibronectin type III “domain-containing” protein 5 (FDNC5) and acts on skeletal muscle, resulting in increased energy expenditure and oxidative metabolism through the induction of metabolic genes (Li et al., 2017). Previous reports demonstrated anti-diabetic effect of irisin through increased energy expenditure in mice (Boström et al., 2012). Moreover, irisin plays a key role on the skeleton by increasing cortical bone mineral density, modifying its structure and improving bone strength (Colaianni et al., 2015). In humans, contradictory effects of exercise in irisin levels have been reported (Moreno-Navarrete et al., 2013; Lopez-Legarrea et al., 2014; Shi et al., 2016). While higher irisin plasma levels after exercise in humans have been described, Timmons et al. (2012) using gene expression arrays, failed to detect a robust and consistent increase in FNDC5 mRNA in human muscle biopsies after exercise. The threshold of exercise required to produce these effects might be crucial, because acute exercise has been demonstrated to result in increased serum irisin concentrations in humans (Huh et al., 2012; Anastasilakis et al., 2014). Lower circulating irisin is a marker of muscle weakness and atrophy and is associated with total muscle mass (Huh et al., 2012; Kim et al., 2015; Chang et al., 2017). Taken together, these data suggest that resistance exercise may result in increased circulating irisin.

Myostatin, also known as growth differentiation factor 8, is a secreted TGF-β superfamily protein that is expressed in skeletal muscle, controlling myoblast proliferation, but being a potent negative regulator of skeletal muscle growth and development (Lee, 2004). Myostatin is synthesized as a 376 amino acid (aa) preprotein that consists of 24 aa signal peptide, a 243 aa propeptide, and a 109 aa mature protein (McPherron et al., 1997). The secreted proprotein is cleaved by BMP-1 family proteases to separate the propeptide from the bioactive mature protein (Lee and McPherron, 2001; Zimmers et al., 2002; Wolfaman et al., 2003). This cleavage results in a latent complex containing a disulfide-linked dimer of the mature protein and two associated propeptides (Lee and McPherron, 2001; Zimmers et al., 2002), in which the myostatin propeptide inhibits the active form.

In the current study, we aimed to investigate the association between resistance exercise and circulating levels of irisin and myostatin in elderly subjects (more than 60 years).

## Materials and Methods

### Subjects’ Recruitment

This is a prospective and controlled clinical trial in which subjects were randomized into 3 groups: (1) control group (*n* = 15), (2) resistance exercise group (RE) (*n* = 14), and (3) resistance exercise nutritional support group (RENS) (*n* = 9) during 16 weeks. To increase the sample size of control group, five additional patients were recruited. Randomization was done with the EPIDAT 4.0 program. The subjects (men and women aged 60–75 years) residing in province of Girona, Spain; users of civic centers and primary health care center were invited to participate. Those who accepted were enrolled after giving written informed consent, being studied at baseline and after 16 weeks. This study excluded subjects who (i) were known to have associated chronic diseases (i.e., type 2 diabetes poorly controlled); (ii) were under treatment that could cause myopathy; and (iii) suffer from dementia, cognitive limitation, respiratory impairment or malnutrition (IMC <17 kg/m2, involuntary weight loss >10% and albumin levels <2.5 g/dl).

The night before the visit, all participants followed their usual diet. The post-intervention visit was performed a week after the last session of exercise. For each patient the following measurements at baseline and at post-intervention visit were carried out: (i) Analysis of the biochemical parameters at fasting state; (ii) Anthropometric measurements: brachial circumference, arm circumference, calf circumference, abdominal circumference, tricipital fold; (iii) Fragility test (Fried et al., 2001); (iv) World Health Organization Quality of Life – BREF (WHOQOL-BREF); (v) Mini nutrition assessment (MNA); (vi) Evaluation of muscle strength with a dynamometer; (vii) Assessment of the physical condition with the Short Physical Performance Battery (SPPB).

### Exercise Program

The exercise program lasted 16 weeks and followed the basic principles of the training: (1) loading principle, (2) progression, (3) specificity and individuality, and (4) recovery [American college of sport medicine position stand (1998)]. The subjects performed two weekly sessions of approximately 45 min, with a minimum day of rest between sessions. The session consisted of 3 parts: (i) Warm up with stretching and joint mobilization exercise; (ii) Main part where the resistance exercises will be worked out: 4 upper body exercises and 3 lower body exercises; (iii) Return to the calm with stretching and relaxation. The first weeks of the program were for the anatomical adaptation and the knowledge for the exercises execution; later and progressively increased the load and the volume. All subjects were directly supervised by the physical educator who was in charge.

### Nutritional Intervention

The individuals in the control group (C) and the resistance exercise group (RE) followed their usual diet. Individuals in the resistance exercise and nutritional support group (RENS) followed a balanced and varied diet. A protein intake of 0.8–1 g of protein per kg of weight was guaranteed, distributed in 3 meals per day (breakfast/lunch/dinner). The protein intake was calculated from three 24-h reminders as previously reported (Wu et al., 2017). Dietary advice was given to follow a diet enriched with proteins and also was provided with powdered protein (calcium caseinate, T. Aliment – Espècies Teixidor, Barcelona, Spain).

#### Nutritional Support

The 10 g of protein (calcium caseinate) powder diluted in water or juice was taken daily at breakfast and twice week additional 10 g of protein administration just after exercise performance.

### Analytical Methods

Serum glucose, glycated hemoglobin (HbA1c), serum insulin, total cholesterol, LDL and HDL cholesterol, triglycerides, were determined as described elsewhere (Moreno-Navarrete et al., 2013). C-reactive protein (ultrasensitive assay; 110 Beckman, Fullerton, CA, United States) was determined by a routine laboratory test. Leucocytes, neutrophils and lymphocytes cell count and albumin and creatinine measurement was performed by routine laboratory analysis. Irisin were determined using Irisin ELISA Kit (Catalog Number RAG018R, BIOVENDOR, Brno, Czechia). According to manufacturer information, this ELISA is specific for the measurement of natural and recombinant irisin in human samples, and it does not cross-react with FNDC4, human adiponectin, human Nampt, human RBP4, human clusterin, human leptin, human vaspin, human GPX3, human resistin, human ACE2, human lipocalin-2, human ANGPTL3, human ANGPTL6, human DNER, human DLK1, human calreticulin, and human IL-33. Myostatin concentrations were measured by Quantikine^®^ELISA GDF-8 / Myostatin Immunoassay (Catalog Number DGDF80, R&D Systems, Inc., Minneapolis, MN, United States). In both irisin and myostatin ELISA, intra- and interassay coefficients of variation were lower than 10%. Irisin was analyzed in all participants from RE and RENS group, but only in first recruited control (*n* = 15) subjects. Otherwise myostatin, which was measured several months after the end of the study, was analyzed in all participants.

### Statistical Analyses

Statistical analyses were performed using SPSS 12.0 software. Unless otherwise stated, descriptive results of continuous variables are expressed as mean and SD for Gaussian variables or median and interquartile range. The relation between variables was analyzed by simple correlation (Spearman’s test). One factor ANOVA with *post hoc* Bonferroni test and paired *t*-test were used to compare categorical parameters. Levels of statistical significance were set at *p* < 0.05.

## Results

The current study included 20 participants (18 women and 2 men) in C, 14 participants (12 women and 2 men) in RE and 9 participants (only women) in RENS, with an age range of 60–75 years-old (Table 1). Mean age was significantly higher in RENS compared to RE group (71.2 ± 3.3 vs. 64.9 ± 5.5, *p* = 0.02) (Table 1).

**TABLE 1:** Evolution of body composition, anthropometric parameters walking speed and strength.

	**Control (*n* = 20)**			**RE (*n* = 14)**			**RENS (*n* = 9)**				
	**At baseline**	**At follow-up**	p intra ^*^	**At baseline**	**At follow-up**	p intra^*^	**At baseline**	**At follow-up**	p intra^*^	**P inter^∗∗^ At baseline**	**P inter^∗∗^ At follow-up**
Age	66.4 (±4.6)			64.9 (±5.5)			71.2 (±3.3)^#∇^			**0.016**	
Weight (kg)	71 (±14.3)	70.7 (±14.7)	0.530	68.9 (±14.2)	68.7 (±14.1)	0.444	72.3 (±11.6)	72.5 (±12)	0.465	0.813	0.800
BMI (kg/m^2^)	28.9 (±4.6)	28.7 (±4.8)	0.231	29.1 (±4.8)	29 (±4.7)	0.505	30 (±2.8)	30.1 (±3.1)	0.525	0.861	0.723
Waist perimeter (cm)	93 (±11.9)	90 (±10.7)	**0.003**	92.8 (±14.4)	92.6 (±14.2)	0.837	96.1 (±9.9^*^)	96.1 (±10.5)	0.809	0.727	0.670
Arm circumference (cm)	31.6 (±4)	31.8 (±4.2)	0.589	31 (±2.3)	31.4 (±3)	0.243	32.2 (±3.3)	32.4 (±3)	0.635	0.675	0.806
Muscle arm circumference (cm)	23.6 (±2.7)	24 (±2.1)	0.443	22.5 (±2)	23.3 (±2.6)	0.088	23.3 (±3)	24.3 (±2)	**0.038**	0.451	0.621
Abdominal circumference (cm)	105 (±11)	105 (±10)	0.664	92 (±10)	84 (±9)	0.287	97.5 (±12.9)	102 (±7.9)	0.231	0.160	**0.040**
Calf circumference (cm)	33.1 (±3)	32 (±2.8)	**0.008**	33 (±2.4)	32.7 (±2.1)	0.504	32.3 (±4.3)	36.4 (±1.3)	**0.025**	0.735	**0.001**
Basal metabolism (kcal)	1372 (±218)	1347 (222)	**0.004**	1307 (±254)	1306 (±259)	0.824	1390 (±208)	1409 (±208)	**0.038**	0.600	0.554
Fat mass (kg)	25.1 (±8.6)	25.9 (±8.8)	**0.043**	24.8 (±8.5)	24.6 (±7.7)	0.763	25.7 (±6.5)	25.1 (±7.1)	0.294	1	0.911
% Fat mass	34.9 (±5.2)	36.1 (±5.6)	**0.005**	36.3 (±6.6)	36.1 (±5.8)	0.665	35.2 (±6)	34.1 (±6.7)	0.111	0.859	0.560
Fat free mass (kg)	46 (±7.5)	45 (±7.7)	**0.002**	43.8 (±9.5)	43.7 (±9.9)	0.733	46.5 (±7.4)	47.4 (±7.4)	**0.046**	0.511	0.583
% Fat free mass	65.4 (±5.6)	64 (±5.2)	**0.003**	63.1 (±6.6)	63.1 (±5.9)	0.909	65 (±6)	65.7 (±6.7)	0.377	0.692	0.5534
Total water (%)	45.4 (±3.4)	44.7 (±3.4)	0.051	45.3 (±4.6)	45.2 (±4)	0.6781	45.1 (±3.9)	456 (±4.5)	0.102	0.739	0.699
Total water (kg)	31.8 (±4.9)	31.1 (±4.9)	**0.016**	30.5 (±6.6)	30.4 (±4)	0.696	32.5 (±5)	32 (±7.9)	0.457	0.369	0.906
Speed at 2.44 m	3.2 (±0.75)	3 (±0.73)	0.082	3.5 (±0.7)	2.9 (±0.5)	**0.005**	3.5 (±0.7)	3.5 (±0.8)	0.881	0.589	0.107
Speed at 4.5 m	5.6 (±1.2)	5.2 (±0.9)	0.134	5.9 (±0.9)	5.2 (±0.1)	**0.013**	5.9 (±1)	5.9 (±1.1)	0.961	0.460	0.140
Speed at 3 m	3.7 (±0.7)	3.4 (±0.7)	**0.016**	3.89 (±0.7)	3.2 (±0.6)	**0.003**	3.9 (0.9)	3.8 (±1)	0.791	0.773	0.210
Grip strength (kg)	16.7 (±4.9)	17 (±5.6)	0.639	15.3 (±4.6)	17.2 (±4.6)	**0.011**	16.8 (±5.9)	17.8 (±6.5)	0.185	0.638	0.946

^*^p-value applying T student for matched data. ^∗∗^*p*-value applying one-way anova. ^#^*p* = 0.03 compared to C. ^∇^*p* = 0.02 compared to RE. Bolded values mean statistical significance (*p* < 0.05).

Changes in body composition, anthropometric and physical condition parameters at baseline and at the end of study in each group were shown in Table 1. At baseline, no significant differences were found between control and intervention groups (Table 1).

**FIGURE 1:**
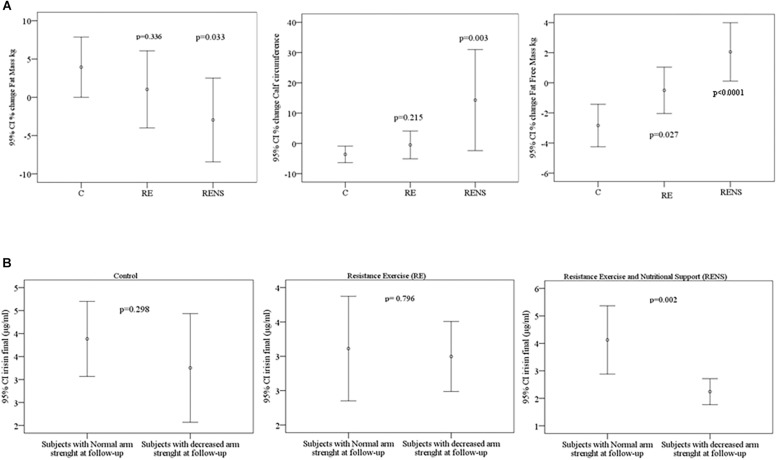
**(A)** Comparison of percent change of fat free mass, calf circumference and fat mass according to control (C), resistance exercise (RE), and resistance exercise plus nutritional support (RENS). **(B)** Comparison of circulating irisin according to arm strength in C, RE, and RENS groups.

Compared to C, RENS resulted in increased percent change of fat free mass kg and calf circumference and in decreased percent change of fat mass kg (Figure 1A). RE also resulted in increased percent change of fat free mass kg compared to C (*p* = 0.027) (Figure 1A).

In C group, a significant decrease in waist and calf circumference, fat free mass, HbA1c, serum myostatin and speed at 3 m, but in the context of increased fat mass was found (Tables 1, 2). In RE group, a significant increase in strength and circulating irisin, and a decrease in speed at 2.44, 4.5, and 3 m was found (Tables 1, 2). In RENS group, a significant increase in muscle arm circumference, calf circumference, fat free mass and circulating irisin was observed (Tables 1, 2). Interestingly, in this group, strength was positively correlated with fat free mass [(*r* = 0.782, *p* = 0.008), Table 3].

**TABLE 2:** Clinical and biochemical parameters in study cohort.

	**Control (*n* = 20)**			**RE (*n* = 14)**			**RENS (*n* = 9)**				
	**At baseline**	**At follow-up**	p intra ^*^	**At baseline**	**At follow-up**	p intra^*^	**At baseline**	**At follow-up**	p intra^*^	**P inter^∗∗^ At baseline**	**P inter^∗∗^ At follow-up**
Glucose (mg/dl)	110 (±33)	109 (±19)	0.878	104 (±26)	102 (±22)	0.613	97 (±10)	98 (±8)	0.557	0.473	0.279
Insulin (μIU/ml)	7.2±15)	7.1 (±4)	0.954	14.1 (±23)	7.6 (±4.8)	0.295	4.4 (±8.7)	8.4 (±5.8)	0.075	0.316	0.793
HbA1c (%)	5.8 (±0.5)	5.7 (±0.5)	**0.008**	5.8 (±0.8)	6 (±0.5)	0.088	5.5 (±0.3)	5.6 (±0.3)	0.399	0.3475	0.634
HOMA	2.8 (±2.5)	2 (±1.4)	0.243	5.3 (±10.7)	2 (±1.6)	0.297	1 (±2.3)	2.1 (±1.6)	0.076	0.297	0.966
Albumin (g/dl)	4.5 (±0.2)	4.4 (±0.2)	**0.043**	4.5 (±0.2)	4.4 (±0.2)	0.497	4.3 (±0.1)	4.2 (±0.1)	0.282	0.052	0.128
Protein (g/dl)	7.1 (±0.3)	7.1 (±0.3)	0.523	7.1 (±0.3)	7 (±0.3)	0.088	6.9 (±0.1)	6.9 (±0.1)	0.255	0.354	0.302
Total cholesterol (mg/dl)	210 (±30)	203 (±30)	0.073	224 (±56)	227 (±68)	0.728	214 (±37)	217 (±42)	0.0624	0.619	0.336
HDL-cholesterol (mg/dl)	65 (±14)	61 (±14)	0.110	61 (14)	62 (±15)	0.805	64 (±16)	63 (±15)	0.566	0.758	0.972
LDL-cholesterol (mg/dl)	125 (±28)	120 (±128)	0.192	141 (±52)	144 (±60)	0.755	130 (±36)	134 (±41)	0.622	0.467	0.266
Triglycerides (mg/dl)	101 (±36)	105 (±38)	0.570	117 (±76)	122 (±94)	0.453	96 (±41)	100 (±47)	0.544	0.592	0.623
C-reactive protein (mg/dl)	2.2 (8.3)	1.5 (±3)	0.642	0.7 (±1.3)	0.8 (±2.4)	0.858	0.3 (±0.2)	0.4 (±0.3)	0.326	0.614	0.533
Leucocytes	6.8 (±2.3)	6.1 (±1.4)	0.128	5.7 (±1.7)	6 (±2)	0.141	5.6 (±0.9)	5.7 (±1)	0.471	0.120	0.821
Neutrophils	3825 (±1910)	3310 (±1042)	0.134	3100 (±1204)	3240 (±1382)	0.348	3211 (±726)	3211 (±765)	0.405	0.289	0.973
Lymphocytes	2059 (±608)	2047 (±587)	0.482	2000 (±633)	1866 (±588)	0.191	1940 (±765)	1760 (±613)	0.301	0.449	0.709
Irisin (μg/ml)^#^	3.1 (±0.9)	3.5 (±1.1)	0.127	2.4 (±0.3)	3.1 (±0.8)	**0.011**	2.6 (±1.3)	3 (±1.1)	**0.030**	0.116	0.407
Myostatin (ng/dl)	2.5 (1.8–3.1)	1.9 (1.5–2.3)	**0.003**	1.37 (1.2–2.2)	2 (1.4–2.5)	0.203	2.3 (2–2.9)	2.1 (1.8–3.4)	0.736	**0.025**	**0.048**

^*^p-value applying T student for matched data. ^∗∗^*p*-value applying one-way anova. **^#^**In control group was analyzed only in 15 participants (as explained in methods), whereas in RE and RENS was analyzed in all participants. Bolded values mean statistical significance (*p* < 0.05).

**TABLE 3:** Bivariate correlations between strength with anthropometric parameters.

	**Strength (at follow-up)**					
	**Control (*n* = 20)**		**RE (*n* = 14)**		**RENS (*n* = 9)**	
	**r**	**p**	**r**	**p**	**r**	**p**
Fat free mass at follow-up (kg)	0.232	0.311	0.413	0.183	0.782	**0.008**
Muscle mass at follow-up (kg)	0.232	0.311	0.413	0.183	0.782	**0.008**
Protein mass at follow-up (kg)	0.122	0.608	0.521	0.101	0.770	**0.009**
Bone at follow-up (kg)	0.141	0.532	0.393	0.165	0.782	**0.008**

*Bold values mean statistical significance (*p* < 0.05).*

Since mean age in RENS was higher than in RE, multivariate regression analysis was performed after adjusting for age. This analysis indicated that fat-free mass (β = 0.850, *p* = 0.004) contributed independently to strength variance at follow up after controlling for age in RENS group.

The study showed a percentage of change in force of 2.9% in C group, of 16% in RE and 4% in RENS (*p* = 0.181). In RE group, the gain of strength expressed in kg significantly increased from an at baseline strength (from 15.3 to 17.2 kg, *p* = 0.011), and in RENS group also tended to increase (from 16.8 to 17.8 kg, *p* = 0.185). Moreover, in RENS group, the force was positively correlated with kg of fat-free mass (*p* = 0.008), kg of muscle mass (*p* = 0.008), kg of bone (*p* = 0.008) and kg protein mass (*p* = 0.009) (Tables 1, 3).

Of note, in RENS group, circulating irisin was significantly decreased in those participants with decreased arm strength at the end of the study (Figure 1B).

No significant correlation between circulating irisin and myostatin in any group was observed (Table 4).

**TABLE 4:** Bivariate correlations between Irisin and Myostatin.

**Control (*n* = 15)**								
	**Irisin**		**Myostatin**		**Irisin**		**Myostatin**	
	**(at baseline) (μg/ml)**		**(at baseline) (ng/ml)**		**(at follow-up) (μg/ml)**		**(at follow-up) (ng/ml)**	
	**r**	**p**	**r**	**p**	**r**	**p**	**r**	**p**
Irisin (at baseline) (μg/ml)	–	–	–0.027	0.928	0.470	0.077	0.333	0.266
Irisin (at follow-up) (μg/ml)	0.470	0.077	0.099	0.748	–	–	–0.041	0.894
RE (*n* = 14)								
Irisin (at baseline) (μg/ml)	–	–	0.057	0.846	–0.013	0.965	–0.537	**0.048**
Irisin (at follow-up) (μg/ml)	–0.013	0.965	0.011	0.970	–	–	0.132	0.653
RENS (*n* = 9)								
Irisin (at baseline) (μg/ml)	–	–	–0.683	0.042	0.833	**0.005**	–0.483	0.187
Irisin (at follow-up) (μg/ml)	0.833	**0.005**	–0.467	0.205	–	–	–0.483	0.187

*Bold values mean statistical significance (*p* < 0.05).*

**FIGURE 2:**
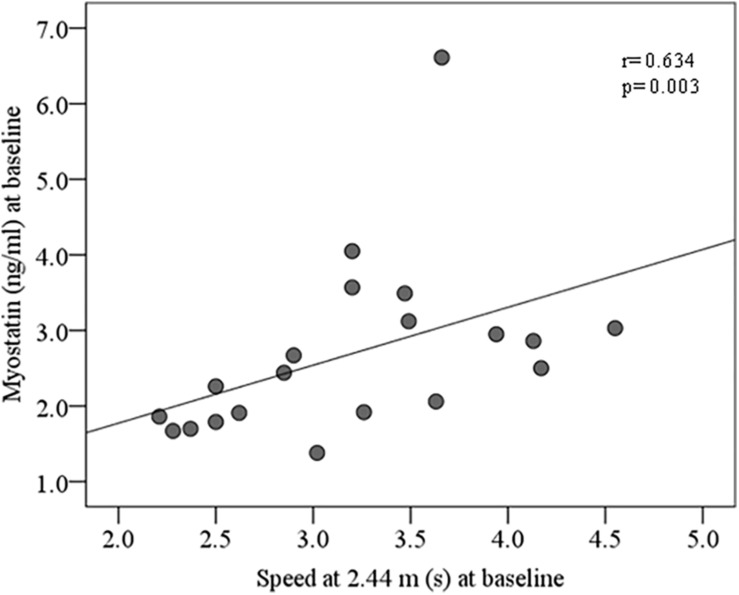
Bivariate correlation between circulating myostatin and speed at 2.44 m at baseline in control group.

At baseline, no significant correlation between circulating irisin and anthropometrical and clinical parameters were observed. Circulating myostatin was positively correlated with weight, muscle arm circumference, basal metabolism, fat-free mass, total water and negatively correlated with C-reactive protein (Table 5). In control group, circulating myostatin was positively correlated with speed at 2.44 m (Figure 2).

**TABLE 5:** Bivariate correlations between anthropometrical and clinical parameters and circulating irisin and myostatin in all participants at baseline.

	**Irisin**		**Myostatin**	
	**r**	**p**	**r**	**p**
Weight (kg)	–0.039	0.809	0.316	**0.039**
BMI (kg/m^2^)	0.009	0.955	0.198	0.203
Arm circumference (cm)	–0.201	0.207	0.285	0.064
Muscle arm circumference (cm)	–0.174	0.276	0.396	**0.009**
Waist perimeter (cm)	0.113	0.506	0.040	0.806
Abdominal circumference (cm)	0.033	0.878	0.255	0.191
Calf circumference (cm)	0.094	0.621	0.180	0.316
Basal metabolism (kcal)	–0.019	0.909	0.365	**0.016**
Fat mass (kg)	–0.070	0.622	0.144	0.357
%Fat mass	–0.128	0.430	0.053	0.741
Fat free mass (kg)	0.044	0.785	0.358	**0.018**
% Fat free mass	0.194	0.224	–0.028	0.858
Total water (kg)	0.042	0.794	0.364	**0.017**
%Total water	0.154	0.336	–0.077	0.623
Speed at 2.44 m (seconds)	–0.018	0.912	0.258	0.095
Speed at 4.55 m (seconds)	0.165	0.310	–0.086	0.588
Speed at 3 m (seconds)	–0.144	0.370	0.106	0.498
Grip strength (kg)	0.264	0.096	–0.048	0.761
Glucose (mg/dl)	–0.036	0.829	–0.071	0.665
Insulina (μIU/ml)	–0.164	0.339	–0.192	0.218
HbA1c (%)	0.146	0.368	–0.181	0.249
HOMA	–0.070	0.698	–0.163	0.316
Albumin (g/dl)	0.153	0.358	0.023	0.890
Protein (g/dl)	–0.102	0.535	–0.197	0.212
Total cholesterol (mg/dl)	–0.044	0.784	–0.107	0.494
HDL cholesterol (mg/dl)	–0.058	0.718	0.008	0.958
LDL cholesterol mg/dl)	–0.107	0.513	–0.008	0.580
Tryglicerides (mg/dl)	0.144	0.369	–0.086	0.582
C-reactive protein (mg/dl)	0.249	0.126	–0.388	**0.012**
Leucocytes	–0.187	0.254	–0.039	0.808
Neutrophils	0.079	0.635	–0.066	0.681
Lymphocytes	–0.159	0.335	–0.200	0.209

*Bold values mean statistical significance (*p* < 0.05).*

At follow up, no significant associations among irisin, anthropometrical and clinical parameters in control (Table 6), RE (Table 7) or RENS (Table 8) were found. In control group, circulating myostatin was positively correlated BMI, abdominal circumference, total water, HOMA, triglycerides, C-reactive protein (Table 6), but these associations were not observed in RE (Table 7) or RENS groups (Table 8).

**TABLE 6:** Bivariate correlations between anthropometrical and clinical parameters and circulating irisin and myostatin in control group at follow-up.

	**Irisin**		**Myostatin**	
	**r**	**p**	**r**	**p**
Weight (kg)	–0.021	0.940	0.370	0.109
BMI (kg/m^2^)	0.002	0.995	0.464	**0.039**
Arm circumference (cm)	–0.013	0.964	0.196	0.422
Muscle arm circumference (cm)	0.143	0.626	0.345	0.148
Waist perimeter (cm)	–0.040	0.893	0.423	0.071
Abdominal circumference (cm)	0.014	0.966	0.487	**0.041**
Calf circumference (cm)	0.353	0.215	–0.107	0.662
Basal metabolism (kcal)	0.011	0.970	0.388	0.091
Fat mass (kg)	–0.103	0.725	0.366	0.123
%Fat mass	–0.263	0.344	0.065	0.784
Fat free mass (kg)	–0.046	0.876	0.337	0.158
% Fat free mass	0.169	0.563	–0.131	0.593
Total water (kg)	0.058	0.851	0.547	**0.019**
%Total water	0.236	0.437	–0.024	0.925
Speed at 2.44 m (seconds)	–0.109	0.699	0.387	0.092
Speed at 4.55 m (seconds)	0.091	0.747	0.239	0.310
Speed at 3 m (seconds)	–0.247	0.375	0.268	0.253
Grip strength (kg)	0.071	0.800	–0.078	0.743
Glucose (mg/dl)	–0.446	0.110	0.297	0.217
Insulina (μIU/ml)	–0.258	0.394	0.308	0.187
HbA1c (%)	–0.201	0.472	0.051	0.829
HOMA	–0.371	0.236	0.508	**0.026**
Albumin (g/dl)	–0.013	0.964	–0.130	0.585
Protein (g/dl)	–0.069	0.816	0.131	0.594
Total cholesterol (mg/dl)	–0.339	0.216	–0.333	0.151
HDL cholesterol (mg/dl)	–0.232	0.405	–0.348	0.133
LDL cholesterol mg/dl)	–0.338	0.218	–0.348	0.133
Tryglicerides (mg/dl)	0.289	0.297	0.462	**0.040**
C-reactive protein (mg/dl)	–0.068	0.810	0.528	**0.017**
Leucocytes	–0.147	0.615	0.120	0.624
Neutrophils	–0.101	0.730	0.227	0.349
Lymphocytes	0.056	0.844	–0.320	0.169

*Bolded values mean statistical significance (*p* < 0.05).*

**TABLE 7:** Bivariate correlations between anthropometrical and clinical parameters and circulating irisin and myostatin in resistance exercise group at follow-up.

	**Irisin**		**Myostatin**	
	**r**	**p**	**r**	**p**
Weight (kg)	0.059	0.835	–0.147	0.615
BMI (kg/m^2^)	–0.157	0.576	0.055	0.852
Arm circumference (cm)	0.122	0.664	–0.283	0.327
Muscle arm circumference (cm)	0.018	0.950	–0.218	0.455
Waist perimeter (cm)	0.030	0.914	–0.081	0.782
Abdominal circumference (cm)	0.500	0.667	–0.500	0.667
Calf circumference (cm)	–0.109	0.711	–0.320	0.287
Basal metabolism (kcal)	0.113	0.689	–0.385	0.175
Fat mass (kg)	–0.027	0.937	0.115	0.751
%Fat mass	–0.122	0.666	0.415	0.140
Fat free mass (kg)	0.172	0.594	–0.391	0.235
% Fat free mass	0.193	0.490	–0.431	0.124
Total water (kg)	0.077	0.821	–0.745	**0.013**
%Total water	0.296	0.377	–0.091	0.803
Speed at 2.44 m (seconds)	0.161	0.566	–0.167	0.568
Speed at 4.55 m (seconds)	0.165	0.558	–0.123	0.675
Speed at 3 m (seconds)	–0.113	0.689	–0.279	0.334
Grip strength (kg)	0.227	0.416	–0.266	0.358
Glucose (mg/dl)	0.065	0.819	–0.651	**0.012**
Insulina (μIU/ml)	–0.134	0.647	0.385	0.175
HbA1c (%)	0.174	0.534	–0.527	0.053
HOMA	–0.147	0.615	0.345	0.227
Albumin (g/dl)	0.045	0.874	–0.070	0.812
Protein (g/dl)	–0.274	0.322	0.372	0.191
Total cholesterol (mg/dl)	–0.441	0.099	0.402	0.154
HDL cholesterol (mg/dl)	–0.213	0.446	0.284	0.325
LDL cholesterol mg/dl)	–0.396	0.161	0.560	**0.046**
Tryglicerides (mg/dl)	–0.133	0.636	–0.009	0.976
C-reactive protein (mg/dl)	–0.184	0.529	0.033	0.915
Leucocytes	–0.214	0.443	0.051	0.864
Neutrophils	0.014	0.960	0.221	0.449
Lymphocytes	–0.432	0.108	–0.150	0.609

*Bold values mean statistical significance (*p* < 0.05).*

**TABLE 8:** Bivariate correlations between anthropometrical and clinical parameters and circulating irisin and myostatin in resistance exercise and nutritional support group at follow-up.

	**Irisin**		**Myostatin**	
	**r**	**p**	**r**	**p**
Weight (kg)	–0.233	0.546	0.517	0.154
BMI (kg/m^2^)	–0.267	0.488	0.150	0.700
Arm circumference (cm)	–0.400	0.286	0.033	0.932
Muscle arm circumference (cm)	–0.517	0.154	0.717	**0.030**
Waist perimeter (cm)	0.133	0.732	0.467	0.205
Abdominal circumference (cm)	–0.143	0.736	0.238	0.570
Calf circumference (cm)	0.554	0.154	–0.289	0.487
Basal metabolism (kcal)	0.183	0.637	0.350	0.356
Fat mass (kg)	–0.250	0.516	0.133	0.732
%Fat mass	–0.350	0.356	–0.150	0.700
Fat free mass (kg)	0.183	0.637	0.350	0.356
% Fat free mass	0.350	0.356	0.150	0.700
Total water (kg)	0.083	0.831	–0.117	0.765
%Total water	0.343	0.366	0.142	0.715
Speed at 2.44 m (seconds)	–0.550	0.125	0.267	0.488
Speed at 4.55 m (seconds)	–0.483	0.187	0.300	0.433
Speed at 3 m (seconds)	–0.583	0.099	0.183	0.637
Grip strength (kg)	0.467	0.205	0.250	0.516
Glucose (mg/dl)	0.633	0.067	–0.133	0.732
Insulina (μIU/ml)	0.450	0.224	–0.150	0.700
HbA1c (%)	0.611	0.081	–0.569	0.110
HOMA	0.450	0.224	–0.150	0.700
Albumin (g/dl)	0.061	0.877	0.087	0.325
Protein (g/dl)	–0.042	0.915	0.244	0.527
Total cholesterol (mg/dl)	0.276	0.472	–0.377	0.318
HDL cholesterol (mg/dl)	–0.159	0.683	–0.460	0.213
LDL cholesterol mg/dl)	0.200	0.606	–0.150	0.700
Tryglicerides (mg/dl)	0.483	0.187	–0.233	0.546
C-reactive protein (mg/dl)	0.310	0.417	–0.870	**0.002**
Leucocytes	0.500	0.207	–0.381	0.352
Neutrophils	0.790	**0.020**	–0.323	0.435
Lymphocytes	–0.211	0.586	–0.270	0.482

*Bold values mean statistical significance (*p* < 0.05).*

The walking speed decreased significantly in C and RE group, moving from 3.75 to 3.4 m/s at the end (*p* = 0.016) in C and from 3.9 m/s to a at follow-up speed of 3.2 m/s (*p* = 0.003) in RE, but not in RENS, in which walking speed change from 3.9 m/s to a at follow-up speed of 3.8 m/s (*p* = 0.791) (Table 2).

## Discussion

In this clinical trial, the impact of low-intensity resistance exercise and nutritional assistance program on body composition, anthropometric parameters, strength, speed of motion and myokines were examined in elderly people.

### Body Composition and Anthropometric Parameters

Current data confirmed that low intensity exercising program and nutritional assistance prevented muscle deterioration in people aged 60–75 years old. Exercise improves muscle mass and contributes to the maintenance of daily life activities (Löllgen et al., 2009). Both aerobic and resistance exercise delay the deterioration of the muscles, but resistance exercise seems the most effective (Visvanathan and Chapman, 2010). Here, we showed that 16-week low intensity resistance program produced significant changes on body composition (fat mass, percent fat mass, fat-free mass, muscle mass kg and the calf circumference). These results were expected and are in agreement with previous studies (Liao et al., 2017), and indicated that low intensity resistance training promotes musculature improvement, is a useful and simple therapeutic approach to prevent sarcopenia, and when was combined with protein supplementation these beneficial effects were enhanced (Liao et al., 2017). However, resistance training presents some limitations (Morgan, 2012; Malafarina et al., 2013; Yu, 2015). For instance, one important limitation was the rapid loss of beneficial effects, such as the gain of muscle mass (in the study the improvement is 0.7% *p* < 0.001) and adherence to exercise. The study’s approach of performing low-intensity resistance exercises aims to inform to the subject about exercise strategies that can be performed at home, since equipment is not required to be able to perform them, and the importance of training as long as possible without causing muscle overload injuries (Johnston et al., 2008).

In control group, waist and calf circumference were reduced in parallel to fat free mass and decreased speed at 3 m, indicating aging-associated body weight reduction (Dzien et al., 2013), muscle mass loss and worsening of physical condition. Elderly associated physical inactivity is known to accelerate muscle mass loss (Evans, 2010). In fact, physical activity reduced the progression of muscle aging and decreased the strength of muscle contraction (Hughes et al., 2004; Zampieri et al., 2015), attenuating sarcopenia in pre- and post-menopausal women (Walsh et al., 2006) and in older men (Szulc et al., 2005). On the other hand, 10 days of bed rest resulted in a significant decreased lean mass and skeletal muscle loss in healthy older adults, even greater than young individuals after 28 days (Kortebein et al., 2007).

Otherwise, the reduction of HbA1c from 5.8 to 5.7 has not clinical relevance.

Here, we found the following effects of resistance training on strength, speed and myokines:

(i) *Strength.* Resistance training is the most effective strategy to increase muscle mass and strength (Leenders et al., 2013; Yoshimura et al., 2017). Being the grip strength is a good indicator of low muscle mass (Lauretani et al., 2003), this parameter detects individuals with low muscle mass that will make them more fragile and with greater risk of disability (Ling et al., 2010; Taekema et al., 2010). We observed that there was no significant association between the concentration of myostatin and muscular force, as concluded by Carvalho et al. (2018).
(ii) *Speed.* It is known that exercise interventions that increase muscle strength are very effective in improving walking speed (Gottlob, 2008). In the present study, the walking speed decreased in all groups producing in improvement of 15% in control group, 14% in Resistance Exercise group, and 7% in Resistance Exercise and Nutritional Support group. Different studies have shown improvements between 3 and 10% (Mendieta et al., 2015), being the results obtained in the current study similar to those reported. The walking speed reflects the health and functionality of the person (Abellan van Kan et al., 2009). This is recommended as a clinical indicator to assess survival. In a meta-analysis published in 2011 in the journal JAMA by Studenski et al. (2011) walking speed within their range of normality behaves as a protective factor against mortality, being the lowest in those individuals with faster walking. A lower driving speed is usually associated with disability, cognitive deterioration, accidental falls, various neurological diseases, cardiopulmonary and orthopedic diseases that contribute to increase mortality rate (Abellan van Kan et al., 2009; Studenski et al., 2011).
(iii) *Myokines.* Myostatin is a hormone that is mainly expressed in the muscle and inhibits the formation of muscle mass. Myostatin is generated as a precursor protein that requires the proteolytic division to release the N-terminal and the C-terminal propeptide. The active form of myostatin is a disulfide dimer attached to the C-terminal fragment (White and LeBrasseur, 2014).
  Myostatin increases with age, although differences in total vs. active form have not been well investigated (Yarasheski et al., 2002). The current study, that analyzed serum myostatin concentrations before and after the resistance exercise program, found results that are contradictory with those reported in the literature. In the control group, circulating myostatin concentration decreased significantly after 16 weeks, in parallel to decreased fat free mass, whereas, in RE and RENS group, myostatin remained unchanged after the exercise, and fat free mass increased. These findings were in hard contrast to previous studies that demonstrated negative effects of myostatin in muscle mass development (McPherron et al., 1997; Lee and McPherron, 2001; Zimmers et al., 2002; Wolfaman et al., 2003; Lee, 2004). Possibly, in control group, a reduction of myostatin causes significant decrease in primary fibers of skeletal muscle, which is associated with increased fatigue susceptibility (Hennebry et al., 2009), indicating physical condition decline. In line with current data, in a recent study low serum myostatin levels have been associated with low skeletal muscle (Peng et al., 2018). Muscular force exercise is known to inhibit local myostatin (Brotto and Abreu, 2012). However, Laksmi et al. (2017) observed increased secretion of myostatin in blood in response to exercise. As, the antibodies used to detect myostatin (current ELISA) did not distinguish between the active or latent form, further research is needed.
  Irisin is a myokine produced by the proteolytic cleavage of the membrane-protein, fibronectin type III “domain-containing” protein-5 (FNDC5) (Boström et al., 2012). It is regulated by peroxisome proliferator activated receptor (PPAR) and peroxisome proliferator activated receptor γ co-activator-1-α (PGC1α). It is suggested that irisin may act in some of the beneficial effects of exercise by inducing the uncoupling protein one (UCP-1), that afterward increases the energy expenditure of white adipocytes, process called “browning.” This process is characterized by a switch of white to beige adipose tissue in response to β-adrenergic stimuli (exposure to cold and physical activity) (Novelle et al., 2013). In humans, the effect of exercise on irisin production has provided contradictory findings (Huh et al., 2012; Lecker et al., 2012; Timmons et al., 2012). Huh et al. (2012) correlated the decrease in the circulating irisin concentration with the loss of muscle mass associated to aging. A recent study by Chang et al. (2017) concluded that a low blood concentration of irisin was a sensitive molecular marker for muscle weakness and atrophy. In post-menopausal women, decreased serum irisin concentration is an independent predictor of sarcopenia (Park et al., 2018). In fact, irisin has been proposed as a molecule that combines beneficial effects for treating osteoporosis and muscular atrophy through its effects restoring bone and preventing muscle wasting (Kim et al., 2015; Colaianni et al., 2017).
  In agreement with these studies, current findings indicated that RE and RENS resulted in increased circulating irisin in parallel to strength and walking speed in elderly subjects. In fact, decreased circulating irisin was significantly associated with strength loss at the end of the study (Figure 1B). Thus, irisin could be a potential biomarker of muscle dysfunction (Chang et al., 2017).

In relation to protein supplementation, several studies indicated that protein supplementation improved the adaptations of resistance exercise (Burke et al., 2001; Candow et al., 2006; Cribb et al., 2006; Hulmi et al., 2009; Volek et al., 2013; Morton et al., 2017). Cribb et al. (2006) reported a 5 kg increase of fat-free mass after 10 weeks of resistance training combined with 45 g/day ingest of milk protein. Burke et al. (2001), Candow et al. (2006) demonstrated that milk protein supplementation during 6 weeks of resistance training promoted an increase of 2.3–2.5 kg of fat-free mass. Hulmi et al. (2009) also reported that supplementation with milk protein and 10 weeks of resistance exercise resulted in an increase of 2.5 kg fat free mass. Volek et al. (2013) reported that supplementation with milk protein from cow or soy in addition with resistance training for 9 months increased by 3.6 and 2.6 kg of fat-free mass, respectively. Morton et al. (2017) suggest that one of the mechanisms responsible for the enhancement of skeletal mass could be explained for the improvement of the anabolism of skeletal muscle. These positive effects of protein supplementation do not have the same effects as isolated supplementation with amino acids (Bird et al., 2006; Vieillevoye et al., 2010; Aguiar et al., 2017). In line with all these studies, current findings confirmed an improvement in fat-free mass in the exercise group with cow milk protein supplementation. Even though, this improvement was not so marked as that described in the literature, probably due to relative low protein supplementation (10 g of protein every day and 20 g during exercise days) did not reach the threshold to promote anabolism of muscle protein in older people (Traylor et al., 2018). An important consideration is that the attachment to nutritional support was not homogeneous in all participants.

Resistance exercise and nutritional support resulted in improved anthropometric parameters (muscle arm circumference, calf circumference, basal metabolism and fat free mass) compared with RE intervention. The improvement in walking speed and grip strength was not so marked in the RENS group compared to RE intervention. These differences could be due to significant increased mean age in RENS compared to RE subjects.

## Conclusion

A program of physical exercise of low intensity resistance, together with a nutritional support, improved the deterioration of the musculature in people aged 60–75. Circulating irisin, but not myostatin, constituted a marker for improved muscle strength after resistance exercise in elderly subjects.

## Ethics Statement

The development of the study was carried out in accordance with the Helsinki Declaration of the World Medical Association on the ethical principles for research in human subjects. The current study followed the ethical principles according to the Organic Law 15/99 (LOPD) and the guideline document of good clinical practices. The protocol was approved by the Ethics Committee of the Dr. Josep Trueta University Hospital of Girona on July 30, 2012 under the code 2012099.

## Author Contributions

CP-F, WR, and JF-R participated in the study design and analysis of data. FC, MS-M, MM, JMM-N, and OR participated in acquisition of data. CP-F, WR, JMM-N, and JF-R participated in interpretation of data. CP-F, FC, and JF-R wrote and edited the manuscript. JMM-N and WR revised the manuscript critically for important intellectual content. All authors participated in final approval of the version to be published.

## Conflict of Interest Statement

The authors declare that the research was conducted in the absence of any commercial or financial relationships that could be construed as a potential conflict of interest.
